# A hybrid unsupervised and supervised learning approach for postictal generalized EEG suppression detection

**DOI:** 10.3389/fninf.2022.1040084

**Published:** 2022-12-19

**Authors:** Xiaojin Li, Yan Huang, Samden D. Lhatoo, Shiqiang Tao, Laura Vilella Bertran, Guo-Qiang Zhang, Licong Cui

**Affiliations:** ^1^Department of Neurology, The University of Texas Health Science Center at Houston, Houston, TX, United States; ^2^Texas Institute for Restorative Neurotechnologies, The University of Texas Health Science Center at Houston, Houston, TX, United States; ^3^School of Biomedical Informatics, The University of Texas Health Science Center at Houston, Houston, TX, United States

**Keywords:** epilepsy, generalized tonic-clonic seizure, postictal generalized EEG suppression, EEG, unsupervised learning, hybrid classifier

## Abstract

Sudden unexpected death of epilepsy (SUDEP) is a catastrophic and fatal complication of epilepsy and is the primary cause of mortality in those who have uncontrolled seizures. While several multifactorial processes have been implicated including cardiac, respiratory, autonomic dysfunction leading to arrhythmia, hypoxia, and cessation of cerebral and brainstem function, the mechanisms underlying SUDEP are not completely understood. Postictal generalized electroencephalogram (EEG) suppression (PGES) is a potential risk marker for SUDEP, as studies have shown that prolonged PGES was significantly associated with a higher risk of SUDEP. Automated PGES detection techniques have been developed to efficiently obtain PGES durations for SUDEP risk assessment. However, real-world data recorded in epilepsy monitoring units (EMUs) may contain high-amplitude signals due to physiological artifacts, such as breathing, muscle, and movement artifacts, making it difficult to determine the end of PGES. In this paper, we present a hybrid approach that combines the benefits of unsupervised and supervised learning for PGES detection using multi-channel EEG recordings. A K-means clustering model is leveraged to group EEG recordings with similar artifact features. We introduce a new learning strategy for training a set of random forest (RF) models based on clustering results to improve PGES detection performance. Our approach achieved a 5-second tolerance-based detection accuracy of 64.92%, a 10-second tolerance-based detection accuracy of 79.85%, and an average predicted time distance of 8.26 seconds with 286 EEG recordings using leave-one-out (LOO) cross-validation. The results demonstrated that our hybrid approach provided better performance compared to other existing approaches.

## 1. Introduction

The disease of epilepsy is characterized by unpredictable seizures that occur recurrently and spontaneously (Fisher et al., 2014). Epilepsy affects approximately one in every 26 adults in the United States (Hesdorffer et al., 2011). In an epileptic seizure, large numbers of brain neurons are involved in an excessive, synchronized, and inappropriate electrical discharge that triggers signs and symptoms (Goldenberg, 2010). An individual experiencing seizures may experience temporary confusion, uncontrolled jerking motions of arms and legs, an inability to speak, or loss of consciousness (Clark and Kruse, 1990). Approximately one-third of epilepsy patients are unable to become seizure-free with currently available treatments, increasing their risk of sudden unexpected death in epilepsy (SUDEP) (Petrucci et al., 2020).

Sudden unexpected death in epilepsy is a catastrophic and fatal complication of epilepsy and is the primary cause of mortality in those who have uncontrolled seizures (Devinsky et al., 2016). It ranks second only to stroke in terms of years of potential life lost due to neurological disease (Thurman et al., 2014). For epilepsy patients who die from SUDEP, no anatomical or toxicological causes of death can be identified at autopsy (Okanari et al., 2020). In epilepsy clinic populations, the incidence of SUDEP ranges between 1.1 and 5.9 per 1,000 patient-years, whereas it is between 6.3 and 9.3 per 1,000 patient-years for those with intractable epilepsy, raising a significant public health concern (Zhao et al., 2021). While several multifactorial processes have been involved including cardiac, respiratory, autonomic dysfunction leading to arrhythmia, hypoxia, and cessation of cerebral and brainstem function, the mechanisms underlying SUDEP are not completely understood (Okanari et al., 2020; Petrucci et al., 2020).

Electrophysiological signals obtained in epilepsy monitoring units (EMUs), such as electroencephalogram (EEG), electrocardiogram (ECG), and electromyography (EMG), are usually used to analyze epileptic seizures (Bertram, 2014). To locate seizures and monitor brain activity between seizures, non-invasive scalp EEG, and invasive intracranial EEG are commonly used (Worrell and Gotman, 2011). For the diagnosis of epilepsy, scalp EEG provides critical information regarding whether the seizure disorder is focal or generalized, idiopathic, or symptomatic, or part of a specific epilepsy syndrome (Smith, 2005), and intracranial EEG is one of the techniques used to localize the seizure onset zone in preparation for surgery (Bertram, 2014). Therefore, EEG is an invaluable tool for diagnosing epilepsy and guiding clinical treatment (Rosenow et al., 2015). It has been widely used to identify biomarkers that can help prevent the development of epilepsy, identify specific regions of the brain that cause epilepsy, and ultimately cure epilepsy through surgery (Staba et al., 2014).

A potential risk marker for SUDEP is the postictal generalized EEG suppression (PGES) (Lhatoo et al., 2010; Wu et al., 2016; Vilella et al., 2019), during which electrical activity is suppressed at the end of a seizure (Grigorovsky et al., 2020). Postictal generalized EEG suppression is defined as diffuse EEG background attenuation (less than 10 μV) in the postictal period (Asadollahi et al., 2018). According to a case-control study by Lhatoo et al. (2010), duration of PGES more than 50 seconds (known as prolonged PGES) was significantly associated with a higher risk of SUDEP.

Based on the definition of PGES, it seems straightforward to identify a period of low-amplitude EEG signals (< 10μV). However, real-world data recorded in EMUs may contain high-amplitude signals due to physiological artifacts such as respiration, muscle, and movement-related artifacts (Li et al., 2020). Therefore, in practice the duration of PGES is determined manually with visual inspection of EEG signal readings by clinical experts, who can leverage additional video recordings along with signals to identify high-amplitude artifacts that are not real EEG activities (Theeranaew et al., 2017). However, such a manual task is time-consuming and labor-intensive, and the judging criteria of PGES with artifacts by each clinical expert are not standardized, which may be subjective and unreliable (Zhao et al., 2021). Automatic PGES detection tools are highly desirable to help clinical experts review and annotate PGES in EEG recordings (Li et al., 2020).

Automated techniques have been studied for PGES detection, including a logistic regression approach based on frequency-domain features (Theeranaew et al., 2017), an eXtreme Gradient Boosting (XGBoost) classifier with time-domain and entropy-based features (Mier et al., 2020), and deep learning models based on convolutional neural network (Kim et al., 2020; Vance et al., 2020). However, these studies utilized segment-based evaluation (i.e., the predictions for each segment determine the performance metrics), which has been demonstrated to be ineffective in measuring the performance of PGES detection in real clinical settings (Li et al., 2020). In a previous study, we introduced a more practically relevant manner (known as recording-based evaluation) to evaluate automated PGES detection methods based on the time distance, which is the time difference between the detected PGES end time and the actual expert-annotated end time (Li et al., 2020). With such time distance-based evaluation metrics, we developed and evaluated a feature-based random forest (RF) approach for automatic PGES detection with multi-channel EEG recordings. However, the performance of our previous approach declined when being applied to a larger dataset for PGES detection, indicating the need of further improvement of our approach. In addition, in our previous work, the categorization of artifact levels (e.g., artifact-free, mild artifact, moderate artifact, and severe artifact) were based on manual review. It is highly desirable to develop automated approaches to group signals with similar levels of artifacts.

In this paper, we present a hybrid approach for PGES detection by combining different learning strategies of unsupervised and supervised learning. We introduce empirical mode decomposition (EMD)-based features and incorporate K-means clustering model to group EEG recordings with similar artifact features. Then we train different RF classifiers (sample-weighted RF) based on the clustering results. To the best of our knowledge, this is the first work combining unsupervised and supervised learning for automatic PGES detection. We apply this approach to a larger dataset and compare its performance with our previous approach as well as support vector machines (SVM) and XGBoost-based approaches.

## 2. Background

### 2.1. Postictal generalized EEG suppression (PGES)

**Figure 1:**
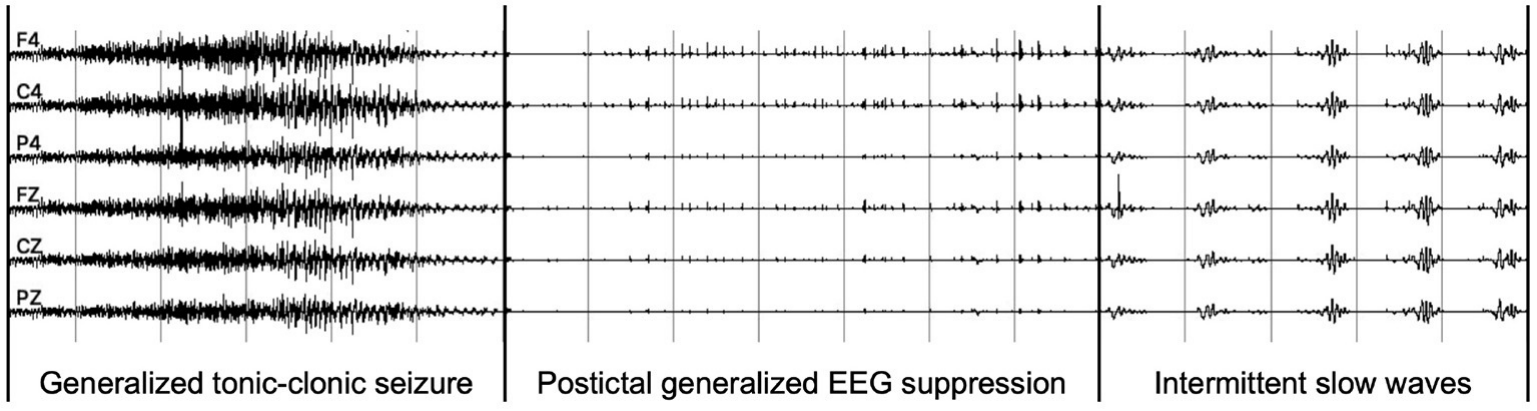
An example of PGES EEG recordings after a generalized tonic-clonic seizure.

The PGES is a postictal generalized attenuation of EEG activity, formerly referred to as a sudden EEG “flattening,” “an abruptly attenuated termination pattern,” or “an electrical shutdown,” and the most commonly used definition now is the one proposed by Lhatoo et al. (Lhatoo et al., 2010; Bruno et al., 2020). Later studies have enhanced this definition by adding additional minimum duration criteria (Surges et al., 2011; Seyal et al., 2012), making it more useful in practice. Postictal generalized EEG suppression mostly occurs following generalized tonic-clonic seizures (GTCS), especially those occurring during sleep, and is associated with postictal immobility, lack of early oxygen administration, duration of oxygen desaturation, and decreased peripheral capillary oxygen saturation nadir values (Alexandre et al., 2015; Kuo et al., 2016; Esmaeili et al., 2018). One example of PGES after a GTCS is shown in Figure 1, and intermittent slow waves (ISW) are the sign of the end of PGES. The Mortality in Epilepsy Monitoring Unit Study (MORTEMUS), which aims to retrieve data from all cardiorespiratory arrests in SUDEP patients to massive brainstem dysfunction, evaluated PGES as a predictor of cardiorespiratory collapse in patients with SUDEP (Yang et al., 2022). Since its discovery, PGES has been of interest in clinical studies investigating other potential markers of SUDEP (Bruno et al., 2020). Therefore, understanding the underlying mechanisms of SUDEP through PGES clinical risk factors is essential for improving risk assessment in epilepsy patients (Yang et al., 2022).

### 2.2. EEG feature extraction

For feature extraction of EEG recordings, we consider the following established features: (1) time-domain features, (2) frequency-domain features, (3) wavelet-based features, (4) inter-channel correlations, and (5) EMD-based features.

*Time-domain features*. Time-domain features include statistical measures (e.g., mean, kurtosis, and skewness) (Jobson, 2012) and Hjorth parameters (Hjorth, 1970). The mean feature measures a probability distribution's central tendency. The kurtosis and skewness features measure the tailedness and the asymmetry of a probability distribution, respectively (Li et al., 2020). Hjorth parameters are generally used in feature extraction for EEG signal analysis (Charbonnier et al., 2011) including activity, mobility, and complexity (Redmond and Heneghan, 2006). The activity measures the variance of a time function. The mobility infers an approximation of the standard deviation of the power spectrum along the frequency axis. The complexity measures the change in frequency, which describes the changes in an EEG recording and how unpredictable those changes can be (Mier et al., 2020).

*Frequency-domain features*. Electroencephalogram recordings have various behaviors in different frequency bands, such as slow-oscillations (0.5–1 Hz), delta bands (1–4 Hz), theta bands (4–8 Hz), alpha bands (8–12 Hz), beta bands (14–30 Hz), and gamma bands (30–80 Hz) (Li et al., 2020). For example, the characteristics of disparate sleep stages in different frequency bands were reported in our previous work (Li et al., 2017). Previous studies demonstrated that spectral power is an important feature for automatic sleep stage scoring and seizure detection (Fraiwan et al., 2012; Li et al., 2019). Therefore, we also regard spectral power in different frequency bands as a feature for PGES detection.

*Wavelet-based features*. The wavelet transform decomposes a signal into a family of wavelets, which are localized in both the time and frequency domains (Mier et al., 2020). It is a relatively recent technique for signal processing compared to the Fourier transform, and the main advantage is that wavelets allow multiresolution analysis in time and frequency simultaneously, which can provide us with the frequency of the signals and the time associated to those frequencies, making it one of the widely used tools for signal analysis and processing (Mallat, 1999; El-Gindy et al., 2021; Omidvar et al., 2021).

*Inter-channel correlations*. Correlation represents the degree of synchrony between two comparing channels and in many aspects presents similar information as cross-coherence analysis of EEG signals (D́ıaz et al., 2015). There have been studies toward finding movement-related information in the patterns of inter-channel connectivity between different brain regions (Gysels and Celka, 2004; Gouy-Pailler et al., 2007; Wei et al., 2007; Grosse-Wentrup, 2008; Chung et al., 2011).

*Empirical mode decomposition*. Empirical mode decomposition is a technique for decomposing a signal without leaving the time domain (Huang et al., 1998). Based on the empirical knowledge of oscillations inherent in a time series, EMD represents these oscillations as a superposition of components having well-defined instantaneous frequencies (Al-Subari et al., 2015). During the EMD process, a given signal is broken down into functions with a mean value of zero and only one extreme between zero crossings, known as intrinsic mode functions (IMFs), which form a complete and nearly orthogonal basis for the original signal (Al-Subari et al., 2015). Furthermore, EMD can reconstruct the original signal by superimposing all extracted IMFs and the remaining slowly changing trends without information loss or distortion (Zeiler et al., 2010).

*Hilbert-Huang transform*. The Hilbert-Huang transform (HHT) uses the EMD method to decompose a signal into IMFs with a trend, and applies the Hilbert spectral analysis (HSA) method to the IMFs to obtain instantaneous frequency data (Oweis and Abdulhay, 2011). Since the IMFs into which a signal is decomposed have the same time domain and length as the original signal, varying frequency over time can be preserved in HHT (Huang et al., 2008). This is an important advantage of HHT since real-world signals often have multiple causes, each of which may happen at specific time intervals (Pachori, 2008). The HHT provides a new method of analyzing non-stationary and non-linear time series data (Aslan and ALçi°n, 2021).

All features were commonly used for feature extraction of EEG signals for detection and prediction of various clinical events, such as sleep scoring, seizure detection, and seizure prediction. Time-domain features, frequency-domain features, wavelet-based features, and inter-channel correlations were also used in previous studies (Kim et al., 2020; Li et al., 2020) for PGES detection. Empirical mode decomposition and wavelet transform both decompose signals into different time-scales. The main difference is that the EMD performs the signal decomposition adaptively and in a data-driven way, whereas the wavelet transform defines a set of pre-fixed filters based on the choice of the mother wavelet (Labate et al., 2013). In EMD, the frequency is obtained by differentiation rather than convolution, which allows to overcome the limitations of the uncertainty principle (Labate et al., 2013). The main advantage of EMD over wavelet transform is the ability to estimate subtle changes in frequency, and EMD-based features have been tested and shown better seizure detection performance compared to wavelet-based features (Kaleem et al., 2021). Empirical mode decomposition's importance in the design of automated detection systems with EEG data is based on the fact that the clinic event gives rise to changes in certain frequency bands. The spectral features obtained from IMF can provide rich clues about the physiology of the EEG signal (Riaz et al., 2015). Therefore, we include EMD-based features in this study, and this is the first time that EMD-based features are used for PGES detection.

### 2.3. K-means clustering and random forest classifier

K-means is one of the simplest and most popular unsupervised machine learning algorithms for clustering (Orhan et al., 2011). By determining K centroids, the K-means algorithm allocates each data point to the nearest cluster, while keeping the within-cluster variances as small as possible.

The RF classifier is an ensemble learning approach, which constructs a number of decision trees to perform classification. The overall output is determined by applying an object to each tree and choosing the classification with the highest voting weight. Misclassification and out-of-bag metrics are used to adjust the weight of each tree (Li et al., 2017). Random forest has been leveraged for detecting various clinic events from EEG recordings (Wei et al., 2020; Abou-Abbas et al., 2021; Dimitriadis et al., 2021; Messaoud and Chavez, 2021).

### 2.4. Evaluation metrics

Validating the performance of a machine learning model is the most important step of the entire workflow, which directly reflects the problem-solving capability of the proposed algorithm and gives quantitative analysis results to determine whether it can be used in real-world scenarios (Li et al., 2021). Evaluation metrics are used to assess the performance of machine learning models. For classification problems, commonly used evaluation metrics including accuracy, precision, sensitivity (as known as recall), specificity, F-score, receiver operating characteristic (ROC), and area under the curve (AUC) (Dalianis, 2018). However, these evaluation metrics in PGES detection can not adequately reflect the actual performance of the model in the clinical practice, i.e., a model with high accuracy, sensitivity, specificity, and F-score (over 90%) may not necessarily achieve completely satisfactory results when deployed in the real clinical scenarios (Li et al., 2020). Therefore, to leverage machine learning-based approaches in PGES detection, we developed a set of time distance and recording-based evaluation metrics in a more clinically relevant way, which were acceptable to clinical experts (Li et al., 2020).

## 3. Methods

### 3.1. Dataset

The EEG data used in this study are obtained from the Center for SUDEP Research (CSR) data repository. Center for SUDEP Research is a Center Without Walls initiative for collaborative epilepsy research supported by the National Institute of Neurological Disorders and Stroke (NINDS). Researchers from 14 universities in the United States and Europe have taken part in the project, bringing extensive and diverse experiences to help better understand SUDEP (Lhatoo et al., 2015, 2016). Center for SUDEP Research aims to better understand cortical, subcortical, and brainstem mechanisms involved in SUDEP through a data-driven, systems biology approach that focuses on cortical influences in SUDEP. The CSR's Informatics and Data Analytics Core (IDAC; NIH U01NS090408) has developed an infrastructure for integrating and analyzing prospectively collected data related to SUDEP from different domains, such as clinical, electrophysiological, biochemical, genetic, and neuropathological fields (Li et al., 2022). The CSR data repository contains multimodal data from over 2,500 epilepsy patients (a broad spectrum of ages as well as social, racial, and ethnic groups), including thousands of 24-hour electrophysiological recordings in the European Data Format (Li et al., 2020).

**Figure 2:**
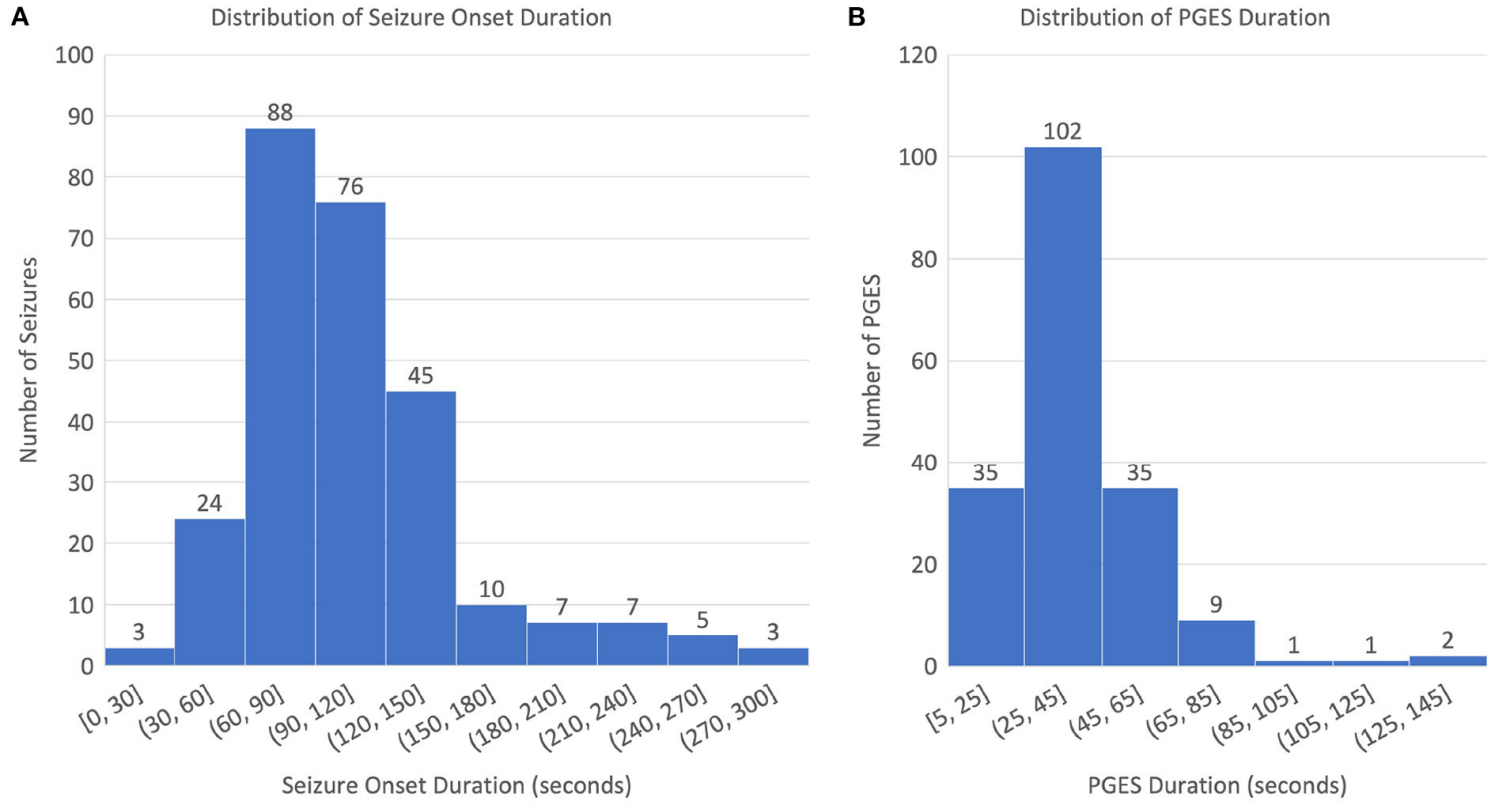
Distributions of seizure onset and PGES: **(A)** distribution of seizure onset duration and **(B)** distribution of PGES duration.

The dataset used for this study consists of 268 EEG recordings from 171 patients (3–81 years old; 76 males, 94 females, and 1 unknown gender; and 4 SUDEP cases) with GTCS in the CSR data repository, with PGES annotated by domain experts. A summary of the dataset, including patient demographics, clinical data, and EEG recording information, can be found in Table 1. The distributions of seizure onset duration and PGES are shown in the Figure 2. We extract five minutes of postictal (i.e, after the end of GTCS) EEG signals for automated PGES detection. A total of 18 EEG channels, which are available to all patients, with a sampling frequency of 200 Hz are utilized: Fp1-F7, F7-T7, T7-P7, P7-O1, Fp2-F8, F8-T8, T8-P8, P8-O2, Fp1-F3, F3-C3, C3-P3, P3-O1, Fp2-F4, F4-C4, C4-P4, P4-O2, Fz-Cz, and Cz-Pz.

**Table 1:** Dataset summary.

Number of patients	171
Age	3–81 years
Gender	Male (76), Female (94), Unknown (1)
Epilepsy classification	Generalized tonic-clonic seizure
Epileptogenic zone	Generalized
Number of SUDEP cases	4
EEG type	Scalp EEG
Number of EEG recordings	268
Number of EEG recordings with PGES	185
Number of EEG recordings without PGES	83
Number of channels	18
Sampling frequency	200 Hz

### 3.2. Hybrid architecture

**Figure 3:**
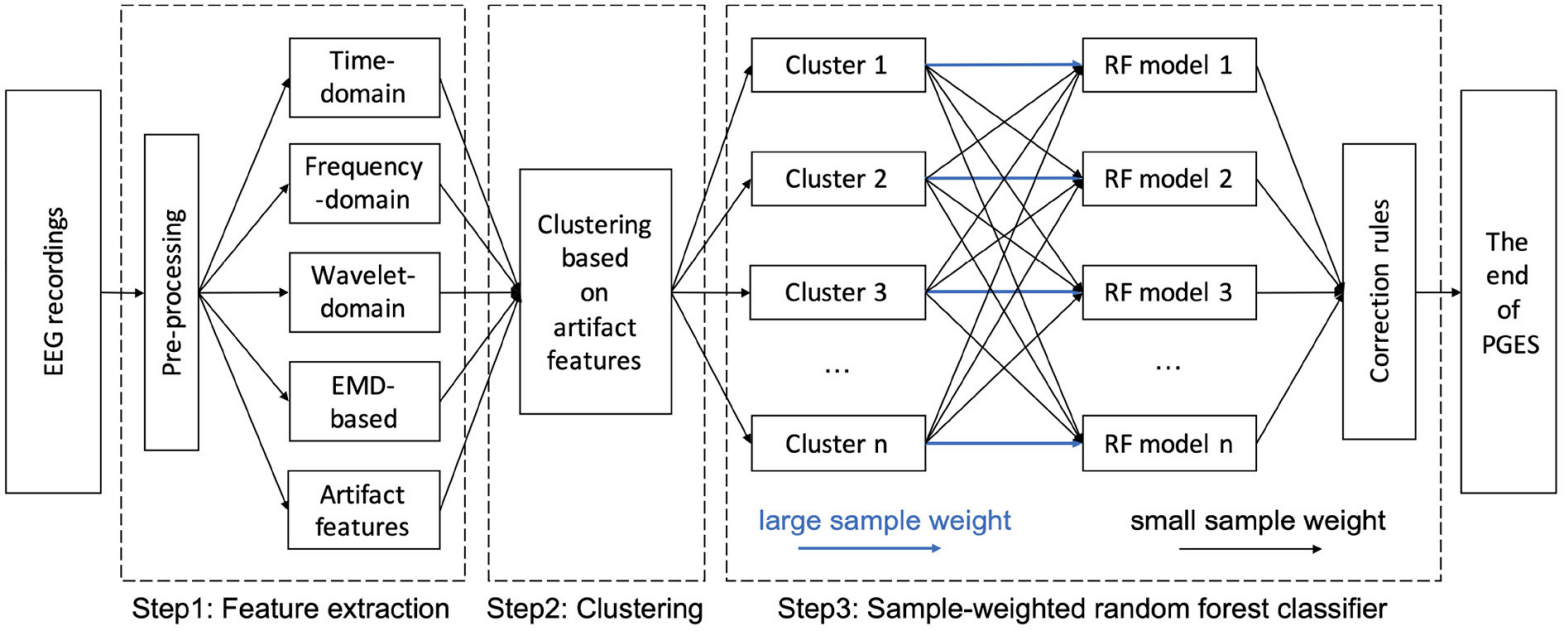
A hybrid architecture for PGES detection.

As shown in Figure 3, the generic architecture for our hybrid approach for PGES detection consists of three steps. The pre-processing and feature extraction of EEG signals start the entire approach (step 1). After feature extraction, K-means clustering are performed to based on the artifact features (step 2). Based on the clustering results, a sample-weighted RF classifier is trained and tested for PGES detection using the extracted features (step 3). Finally, the performance of our approach is evaluated.

### 3.3. Pre-processing and feature extraction

The common electrophysiological artifacts present in EEG recordings include muscle artifacts, breathing, and body and bed movements (Koley and Dey, 2012). To minimize the presence of residual artifacts, the signals are filtered with a band-pass filter with different cutoff frequencies for extracting different types of features: 0.5–30 Hz was used for EMD-based features, and 0.5–5 Hz was used for other features (e.g., time-domain, frequency-domain, wavelet-based features, and inter-channel correlations). The use of filter from 0.5 to 5 Hz was to focus on extracting features in the low-frequency band, where the intermittent slows are located. Each postictal EEG recording is split into signal segments with a length of 1 second (i.e., 1-second signal epoch) from the beginning to the end without any overlap.

For each signal epoch of the 18 EEG channels, we extract all the features utilized in our previous study (Li et al., 2020), including time-domain, frequency-domain, wavelet-domain features, and inter-channel correlations. In addition, we apply EMD and HHT analysis to obtain features that may be hidden in the Fourier domain or in the wavelet coefficients, especially for dynamic or non-sinusoidal signals (Al-Subari et al., 2015). The EMD algorithm decomposes, via an iterative sifting process, a signal *x*(*t*) into *N*-empirical modes *IMF*_*i*_(*t*)(*i* = 1, …, *N*) and a residual *r*_*N*_(*t*):


(1) $$\begin{array}{l} x\left(t\right)=\overset{N}{\underset{i=1}{\sum }}IMF_{i}\left(t\right)+r_{N}\left(t\right). \end{array}$$

Here, an IMF is defined to be a function with the following requirements: (1) the number of local extrema (i.e., the total number of local minima and local maxima) and the number of zero-crossings must either be equal or differ at most by one; and (2) the mean value of the upper and lower envelopes constructed from the local extrema is zero. The procedure of extracting an IMF is called sifting. The sifting process of the signal *x*(*t*) contains the following steps:

1. Find the local minima and maxima of *x*(*t*);
2. Use the local extrema to construct lower and upper envelopes *s*_−_(*t*) and *s*_+_(*t*) of *x*(*t*), and the mean of the envelopes as *m*(*t*) = (*s*_−_(*t*) + *s*_+_(*t*))/2;
3. Subtract the mean from *x*(*t*) to obtain the residual: *y*(*t*) = *x*(*t*) − *m*(*t*);
4. Decide whether *y*(*t*) is an IMF or not by checking the two requirements as described above; and
5. If not, repeat step 1 to step 4 using *y*(*t*) as new *x*(*t*) and end when an IMF is obtained.

**Figure 4:**
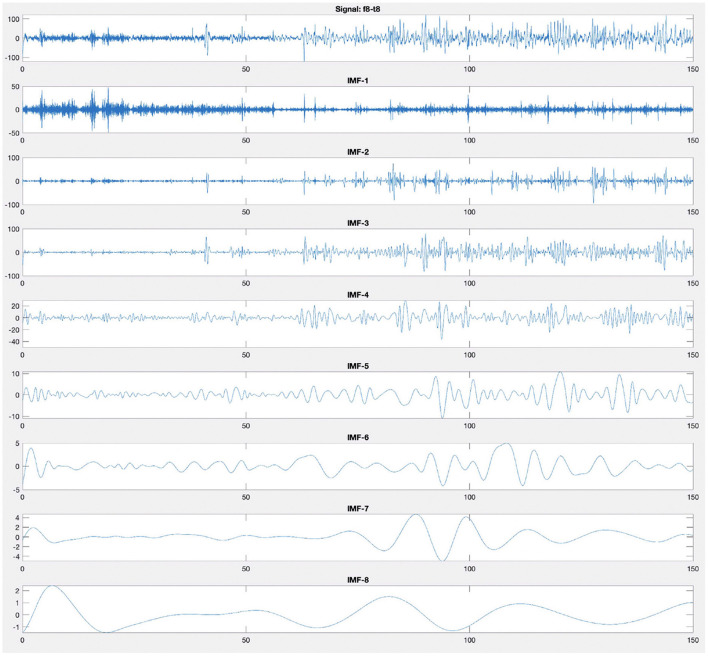
Empirical mode decomposition components of an EEG signal. The first time series is the original EEG recording from F8–T8 channel. The decomposition yields eight IMFs. The IMF are the time frequency constituents or components of the EEG signal. Frequency content is ordered in a descending order (IMF-1 has the highest frequency content).

After calculating the first IMF, *IMF*_1_(*t*), the rest of the signal *r*_1_(*t*) = *x*(*t*) − *IMF*_1_(*t*), which still contains longer period variations in the signal, is treated as the new signal *x*(*t*) and subjected to the same sifting process as described above. The sifting process finally stops when the residue *r*_*N*_(*t*) becomes a monotonic function from which no more IMF can be extracted. Figure 4 illustrates an example of decomposition performed by EMD of an EEG recording from F8–T8 channel. Figure 4 shows that the first mode has a higher frequency than the second mode, and the modes are arranged from the highest frequency to the lowest frequency.

Having obtained the IMF components, the instantaneous frequency can be computed using the Hilbert transform. The final result is a frequency-time distribution of signal amplitude, designated as the Hilbert spectrum, which permits the identification of localized features (Huang et al., 2008). We use the sum of the amplitude of Hilbert spectrum as the feature of each 1-second epoch of the different channels in classification (Step 3 in Figure 3). The main challenge for PGES detection is to discriminate between low frequency artifacts and ISW. The incorrect identification of low frequency artifact as ISW is the main factor causing false positives in PGES detection (Li et al., 2020). Therefore, we extract the amplitudes of the low-frequency portion (0.5–5 Hz) of the Hilbert spectrum of entire 5-minute signal as the artifact features, and apply a clustering approach based on these features (Step 2 in Figure 3) to group EEG recordings with similar low-frequency patterns for reinforcement in later model learning phase.

### 3.4. K-means clustering based on artifact features

Artifacts in EEG recordings are the major challenge for PGES detection because they may lead to false detection of ISW. In our previous study (Li et al., 2020), we manually grouped EEG recordings into different artifact levels, such as artifact-free, mild, moderate, and severe; however, such manual work was time-consuming, labor-intensive, and not scalable. In this work, we use unsupervised learning to perform a clustering analysis to group EEG recordings before feeding the features into the classification model. Through clustering, EEG recordings with similar artifact features are grouped together to reduce the probability of obtaining false positives when performing supervised learning later, thereby improving the performance of PGES detection.

We utilize K-means clustering algorithm, which is an iterative algorithm that attempts to partition the EEG recordings into K pre-defined distinct non-overlapping subgroups (clusters), where each EEG recording belongs to only one group. K-means clustering tries to make the features within the clusters as similar as possible while also keeping the clusters as different as possible. It assigns EEG recordings to a cluster such that the sum of the squared distance between the features and the cluster's centroid (arithmetic mean of all the features of signals that belong to that cluster) is at the minimum. The less variation we have within clusters, the more similar the EEG recordings are within the same cluster.

### 3.5. Sample-weighted random forest (SWRF)

Random forest consists of a large number of individual decision trees that operate as an ensemble. Each individual tree in the RF spits out a class prediction and the class with the most votes becomes the model's prediction. The intuition behind the RF model is that a large number of relatively uncorrelated models (i.e., individual decision trees) operating as a committee will outperform any of the individual constituent model (Breiman, 2001). Five steps to build the RF with the technique of bootstrap aggregating (bagging) has been detailed described in previous study (Li et al., 2017).

In this work, based on the K-means clustering results, we further train the SWRF models by applying disparate training strategies with different clusters. For example, as shown in Step 3 of Figure 3, when training model 1, the sample weights of the signal features from cluster 1 will be increased, while the signal features in other clusters remain the same. In the SWRF model, the sample weights increase the probability estimates in the probability array, thus affecting the impurity measure in each node and how the feature space is sliced and diced for classification. In this way, it changes the way the nodes are divided and the tree is constructed so that the trained model is more inclined to higher weighted samples, i.e., the trained model pays more attention to higher weighted samples during the learning process. Therefore, the trained model 1 has a higher discrimination ability to make correct decisions on EEG recordings with similar artifact features to cluster 1. Thus, we train and obtain *n* cluster-oriented SWRF models focusing on different clusters. When new data are encountered, we first determine which cluster the new data belonged to, and then applied the corresponding SWRF model for PGES/ISW classification. After the classification step, we apply confidence-based correction rules introduced in our previous study (Li et al., 2020) to correct potential misclassifications caused by sudden PGES/ISW state changes that are unlikely to happen.

### 3.6. Evaluation method

For the PGES detection in practical settings, the predication result of the onset of the first ISW in a given EEG recording (i.e., recording-based) is more important since it indicates the end of PGES, and thus the traditional way of perform segment-based evaluation may not reflect the real performance of the PGES detection methods (Li et al., 2020). Therefore, we leverage the time distance and recording-based evaluation metrics for PGES detection proposed in our previous work (Li et al., 2020), including predicted time distance *TD*_*r*_, 5-second tolerance-based detection accuracy *Acc*_5*s*_, and 10-second tolerance-based detection accuracy rate *Acc*_10*s*_. Given a collection *R* = {*r*_1_, …, *r*_*n*_} of *n* EEG recordings, these metrics are defined as follows:


(2) $$TD_{r_{i}}=\left|P_{end_{i}}-T_{end_{i}}\right|(i=1,\ldots ,n)$$

(3) $$\begin{array}{l} TD_{avg}=\frac{1}{n}\overset{n}{\underset{i=1}{\sum }}TD_{r_{i}} \end{array}$$

(4) $$\begin{array}{l} Acc_{5s}=\frac{\left|r_{i}\in R|TD_{r_{i}}\leq 5s\right|}{n} \end{array}$$

(5) $$\begin{array}{l} Acc_{10s}=\frac{\left|r_{i}\in R|TD_{r_{i}}\leq 10s\right|}{n} \end{array}$$

where, given an EEG recording *r*_*i*_, *P*_*en*_*d*__*i*__ is the predicted end time of PGES (or the predicted time of the first ISW) obtained by the detection method and *T*_*en*_*d*__*i*__ is the actual end time of PGES (or the actual time of the first ISW) according to the expert annotations. *TD*_*avg*_ is the average predicted time distance for all EEG recordings. *Acc*_5*s*_ is the number of EEG recordings whose predicted time distances are within 5 seconds divided by the total number of EEG recordings. *Acc*_10*s*_ is the number of EEG recordings whose predicted time distances are within 10 seconds divided by the total number of EEG recordings.

## 4. Results

### 4.1. Clustering of artifact features

**Figure 5:**
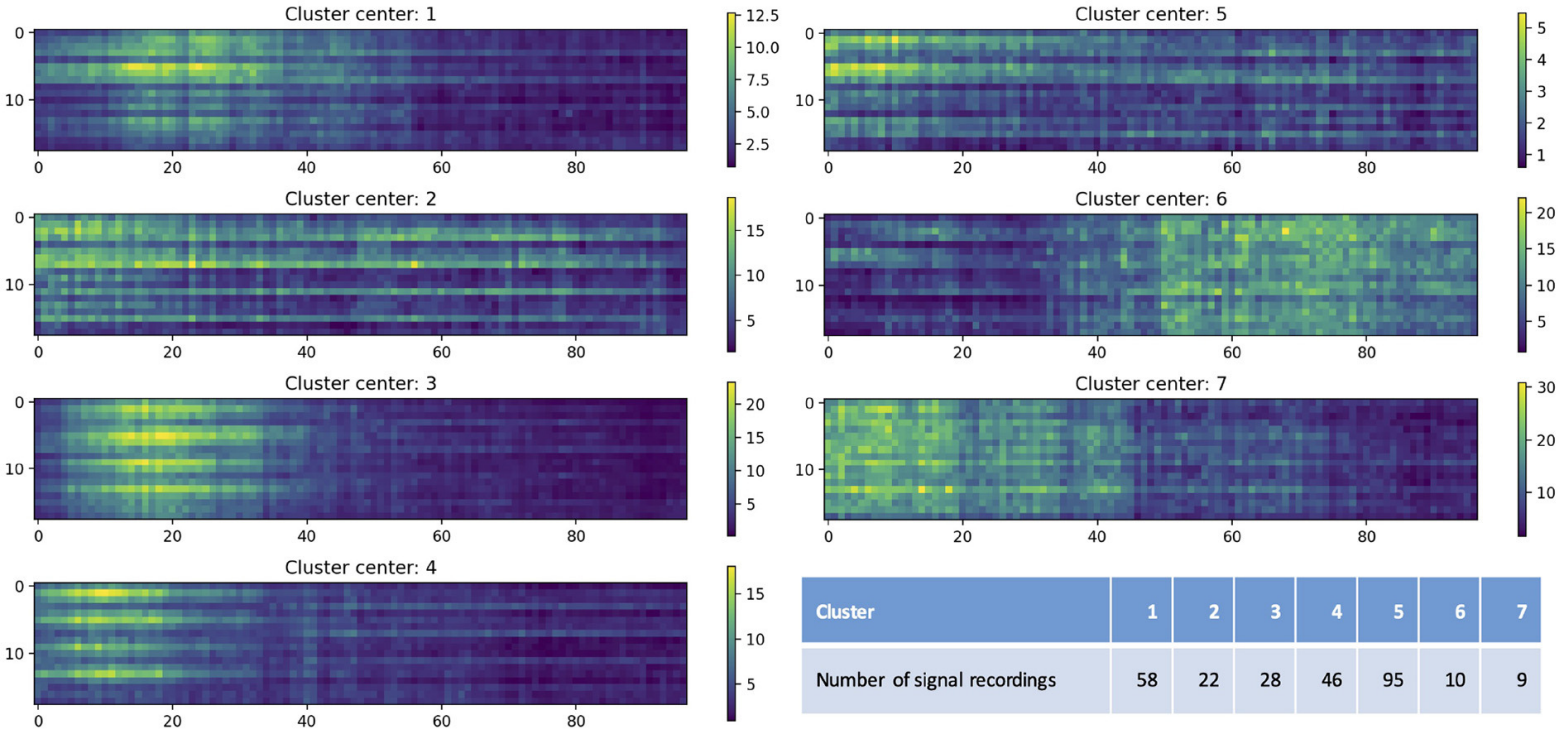
Cluster centers obtained by the K-means clustering algorithm. The x-axis indicates the time after the end of the seizure (in seconds; only shows the first 100 seconds) and y-axis represents the channels (1–18 represent Fp1-F7, F7-T7, T7-P7, P7-O1, Fp2-F8, F8-T8, T8-P8, P8-O2, Fp1-F3, F3-C3, C3-P3, P3-O1, Fp2-F4, F4-C4, C4-P4, P4-O2, Fz-Cz, and Cz-Pz). Colors indicate the value of the artifact features. The table lists the number of EEG recordings in each cluster.

Figure 5 shows the centers of seven clusters obtained by the K-means clustering algorithm with the values of artifact features color-coded, as well as the number of EEG recordings in each cluster. The x-axis indicates the time after the end of the seizure (in seconds; only shows the first 100 seconds) and y-axis represents the channels. Colors indicate the value of the artifact features. The different clusters shown in Figure 5 illustrate varying distributions of artifacts over time periods, with bright yellow indicating more artifacts while dark blue indicating fewer artifacts. For example, for EEG recordings in cluster 3, artifacts were more concentrated between 10 and 20 seconds after the end of the seizure; and for those in cluster 6, artifacts mainly occurred after 50 seconds. It also shows the distribution of artifacts in 18 different channels. For example, in cluster 1, most of artifacts occurred in Fp1-F7, F7-T7, T7-P7, P7-O1, Fp2-F8, F8-T8, T8-P8, and P8-O2; while in cluster 7, artifacts were observed in all 18 channels.

### 4.2. Leave-one-out cross-validation

For the performance evaluation of our hybrid PGES detection method, we used leave-one-out (LOO) cross-validation, which was a special case of cross-validation where the number of folds equals the number of instances in the dataset. Thus, the PGES detection method was applied once for each EEG recording, using all other EEG recordings as a training set and using the given EEG recording as a single-item testing set. For the given EEG recording, we first checked which cluster it belongs to, trained the SWRF model with different sample weights, and then obtained the detection result for this EEG recording. Ultimately, the average predicted time distance (*TD*_*avg*_), 5-second tolerance-based detection accuracy (*Acc*_5*s*_), and 10-second tolerance-based detection accuracy rate (*Acc*_10*s*_) were calculated as the final performance metrics using the detection results of all 268 EEG recordings.

In addition, we experimented with different approaches and compared their performance. These approaches included:

- Our previous PGES detection method (Li et al., 2020), which only used features (referred to as *baseline-features*) including time-domain features, frequency-domain features, wavelet-domain features, and inter-channel correlations and used RF as the classifier. We considered this method as the baseline for the overall comparison.
- Empirical mode decomposition feature based approach, which utilized baseline-features and EMD-based features and applied RF as the classifier.
- Our hybrid approach, which included baseline-features and EMD-based features, and used K-means clustering and SWRF classifiers. We tested different number of clusters (i.e., the value of K): 7, 20, 50, and 100.
- Two additional supervised learning classifiers: support vector machines (SVM) (Kotsiantis, 2007) and XGBoost (Chen et al., 2015).

Table 2 shows the performance evaluation results of different approaches. Compared to the baseline approach, adding EMD-based features improved the PGES detection performance: *Acc*_5*s*_ increased from 56.92% to 63.05%, *Acc*_10*s*_ increased from 70.38% to 77.61%, and *TD*_*avg*_ decreased from 9.47 to 8.85 seconds. With unsupervised learning, the performance was also improved: *Acc*_5*s*_ from 63.05% to 64.92%, *Acc*_10*s*_ from 77.61% to 79.85%, and *TD*_*avg*_ from 8.85 to 8.26 seconds. The results of different number of clusters indicated that the selection of K has a limited impact on overall performance. The results fluctuated considerably when alternative classifiers were applied, and RF classifiers had better performance than both SVM and XGBoost. In general, our hybrid approach provided the best PGES detection performance, which was significantly better than the baseline model: *Acc*_5*s*_ increased from 56.92% to 64.92%, *Acc*_10*s*_ increased from 70.38% to 79.85%, and *TD*_*avg*_ decreased from 9.47 to 8.26 seconds.

**Table 2:** The evaluation results of PGES detection with different approaches.

**Approach**	***Acc*_5*s*_** **(%)**	***Acc*_10*s*_** **(%)**	***TD*_*avg*_** **(seconds)**
Baseline-features + RF (*as baseline*)	56.92	70.38	9.47
Baseline-features + EMD-based features + RF	63.05	77.61	8.85
Baseline-features + EMD-based features + K-means (7) + SWRF	64.18	79.10	8.47
Baseline-features + EMD-based features + K-means (20) + SWRF	64.55	**79.85**	8.46
Baseline-features + EMD-based features + K-means (50) + SWRF	**64.92**	**79.85**	**8.26**
Baseline-features + EMD-based features + K-means (100) + SWRF	63.43	77.61	8.48
Baseline-features + EMD-based features + K-means (7) + SVM	39.18	50.00	19.12
Baseline-features + EMD-based features + K-means (7) + XGBoost	62.31	71.64	10.58

The best performance of each evaluation metric is highlighted in bold.

## 5. Discussion

In this work, we developed a hybrid approach for automated PGES detection based on multi-channel EEG recordings. This hybrid approach combined an unsupervised learning method (K-means clustering) and a supervised learning method (sample-weighted RF). The main idea of our approach is to leverage different learning strategies to improve the PGES detection performance by assigning different weights to each cluster consisting of similar EEG recordings. We evaluated the performance of our approach using the LOO cross-validation method with 268 EEG recordings.

This work has several major distinctions compared with our previous study (Li et al., 2020):

- The new dataset used in this work is larger and more diverse, with the number of EEG recordings increased from 116 to 268 and the number of patients increased from 84 to 171 compared to the previous dataset. Therefore, our hybrid approach in this work has higher levels of generalizability and reliability.
- Previously, we only used 8 EEG channels (i.e., Fp1-F7, F7-T7, T7-P7, Fp2-F8, F8-T8, T8-P8, Fz-Cz, and Cz-Pz) for PGES detection. In this work, we have incorporated 10 more channels, including P7-O1, P8-O2, Fp1-F3, F3-C3, C3-P3, P3-O1, Fp2-F4, F4-C4, C4-P4, and P4-O2. The additional channels provide more information/features on brain activities.
- In this work, we leveraged new EMD-based features, which were not considered in the previous study. The EMD analysis can obtain the signal patterns hidden in the Fourier and wavelet transforms and thus extract the signal features that are different from the other transforms. The evaluation results indicated a significant improvement in PGES detection performance by combining baseline features and EMD-based features.
- Distinct from the previous manual process of differentiating artifact levels of EEG recordings, in this work we automatically extracted artifact features using EMD-based analysis and used unsupervised learning to cluster EEG recordings based on the extracted artifact features. Thus, EEG recordings with similar artifact features were grouped into the same cluster, then different weights were assigned based on the clustering results during the RF classifier learning process. The intuition behind our new approach is to train and predict with similar EEG recordings, avoiding the time-consuming and labor-intensive work of manual artifact level differentiation. The evaluation results demonstrated that the new approach had improved the overall performance.
- In this work, we tested and compared different classification algorithms for performing PGES detection (see Table 2). The results demonstrated that the SWRF model achieved the best performance. Moreover, SWRF had a significant advantage in terms of execution time compared with XGBoost and SVM. Leave-one-out cross-validation is a very time-consuming process when the dataset is large. In terms of execution time, XGBoost took two times longer than SWRF, while SVM spent more (seven times longer than SWRF), which verified the scalability of SWRF.

Automatic detection of PGES is a newly proposed research topic since 2017, and there have been a limited number of published studies on this topic using machine learning methods (Kim et al., 2020; Lamichhane et al., 2020; Zhu et al., 2020). Compared to existing studies, this study used a larger dataset including more patients and EEG recordings, more EEG channels, a hybrid supervised and unsupervised model, as well as an evaluation strategy that is more consistent with clinical practice. In this evaluation strategy, the model is applied and tested on continuous EEG recordings instead of individual signal segments, and the evaluation metrics are more acceptable to clinical experts (Li et al., 2020). Leveraging such clinically relevant evaluation approach, the results can more realistically reflect the performance of the model and provide an accurate reference for applications in practical scenarios.

## 6. Limitations

**Figure 6:**
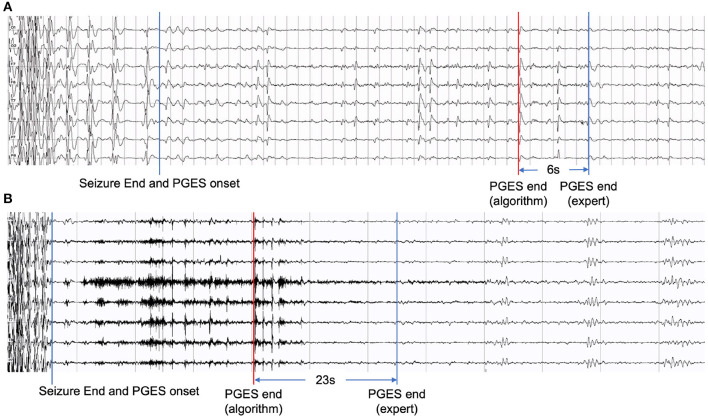
Examples of EEG recordings with false positives: **(A)** with a time distance of 6 seconds and **(B)** with a time distance of 23 seconds.

Although our hybrid approach in this work has shown performance improvement compared with our previous work and other approaches, artifacts, including movement, muscle, unknown, mixed artifacts (combining different kinds of artifacts), remain a major challenge causing false positives in the PGES detection process. Figure 6 shows two examples of EEG recordings with false positives. The signal segments marked in red are misclassified by the algorithm, which resulted in a time distance of 6 seconds (Figure 6A) and 23 seconds (Figure 6B). The misclassified part in Figure 6A was verified by domain experts and confirmed as breath artifacts and the one in Figure 6B was mixed artifacts. In certain scenarios, even for clinicians, it can be difficult to distinguish between artifacts and true brain activities. In future work, we plan to investigate additional artifact-related features to identify artifacts and reduce false positives.

Another challenge is the inter-patient variability, which may affect the performance of the classification algorithm. As the amount of data grows, we plan to develop individual-specific PGES detection methods based on historical patient EEG data. To take full advantage of the increasing amount of data, we also plan to develop deep learning-based methods and compare their PGES detection performances with our hybrid approach in this work.

## 7. Conclusion

In this paper, we presented a hybrid approach combining the benefits of unsupervised and supervised learning for PGES detection based on multi-channel EEG recordings. We incorporated new EMD-based features, which provided valuable information to characterize PGES and ISW. K-means clustering model was leveraged to group EEG recordings with similar artifact characteristics. We introduced a new learning strategy for training a set of RF models according to clustering results to improve the PGES detection performance. The LOO cross-validation results with a total of 286 EEG recordings showed that our method achieved a 5-second tolerance-based detection accuracy of 64.92%, a 10-second tolerance-based detection accuracy of 79.85%, and an average predicted time distance of 8.26 seconds. Comparison of different approaches applied to this dataset of EEG recordings demonstrated that our hybrid approach outperformed others. However, further work toward better handling of artifacts is needed for better performance of automated detection of PGES.

## Data availability statement

The raw data supporting the conclusions of this article is not publicly available. Requests to access the datasets should be directed to the corresponding authors.

## Ethics statement

The studies involving human participants were reviewed and approved by the University of Texas Health Science Center at Houston. Written informed consent to participate in this study was provided by the participants' legal guardian/next of kin.

## Author contributions

G-QZ and LC conceptualized this study. XL, YH, SL, ST, and LVB created the PGES dataset. XL developed the approach with contributions from G-QZ, LC, YH, and LVB. XL and LC wrote and refined the manuscript with contributions from G-QZ, YH, SL, ST, and LVB. All authors contributed to the article and approved the submitted version.

## References

[B1] Abou-Abbas L. Jemal I. Henni K. Mitiche A. Mezghani N. (2021). “Focal and generalized seizures distinction by rebalancing class data and random forest classification,” in International Conference on Bioengineering and Biomedical Signal and Image Processing. BIOMESIP 2021. Lecture Notes in Computer Science, Vol. 12940, eds I. Rojas, D. Castillo-Secilla, L.J. Herrera, and H. Pomares (Cham: Springer), 63–70. 10.1007/978-3-030-88163-4_6

[B2] Alexandre V. Mercedes B. Valton L. Maillard L. Bartolomei F. Szurhaj W.. (2015). Risk factors of postictal generalized EEG suppression in generalized convulsive seizures. Neurology 85, 1598–1603. 10.1212/WNL.0000000000001949 26333799

[B3] Al-Subari K. Al-Baddai S. Tomé A. M. Volberg G. Hammwöhner R. Lang E. W. (2015). Ensemble empirical mode decomposition analysis of EEG data collected during a contour integration task. PLoS ONE 10:e0119489. 10.1371/journal.pone.0119489 25910061 PMC4409116

[B4] Asadollahi M. Noorbakhsh M. Simani L. Ramezani M. Gharagozli K. (2018). Two predictors of postictal generalized EEG suppression: tonic phase duration and postictal immobility period. Seizure 61, 135–138. 10.1016/j.seizure.2018.08.009 30142618

[B5] Aslan M. Alçi°n Z. M. (2021). Detection of epileptic seizures from EEG signals with Hilbert Huang transformation. Cumhuriyet Sci. J. 42, 508–514. 10.17776/csj.682734

[B6] Bertram E. H. (2014). Electrophysiology in epilepsy surgery: roles and limitations. Ann. Ind. Acad. Neurol. 17(Suppl 1):S40. 10.4103/0972-2327.128649 24791088 PMC4001233

[B7] Breiman L. (2001). Random forests. Mach. Learn. 45, 5–32. 10.1023/A:1010933404324

[B8] Bruno E. Richardson M. P. Consortium R.-C. (2020). Postictal generalized EEG suppression and postictal immobility: what do we know? Epileptic Disord. 22, 245–251. 10.1684/epd.2020.1158 32540795

[B9] Charbonnier S. Zoubek L. Lesecq S. Chapotot F. (2011). Self-evaluated automatic classifier as a decision-support tool for sleep/wake staging. Comput. Biol. Med. 41, 380–389. 10.1016/j.compbiomed.2011.04.001 21497802

[B10] Chen T. He T. Benesty M. Khotilovich V. Tang Y. Cho H.. (2015). **** *Xgboost: Extreme Gradient Boosting*. R Package Version 0.4-2. 1:1–4.

[B11] Chung Y. G. Kim M.-K. Kim S.-P. (2011). “Inter-channel connectivity of motor imagery EEG signals for a noninvasive BCI application,” in 2011 IEEE International Workshop on Pattern Recognition in NeuroImaging (Seoul), 49–52. 10.1109/PRNI.2011.9

[B12] Clark V. L. Kruse J. A. (1990). Clinical methods: the history, physical, and laboratory examinations. JAMA 264, 2808–2809. 10.1001/jama.1990.03450210108045 21250045

[B13] Dalianis H. (2018). “Evaluation metrics and evaluation,” in Clinical Text Mining, ed H. Dalianis (Cham: Springer), 45–53. 10.1007/978-3-319-78503-5_6

[B14] Devinsky O. Hesdorffer D. C. Thurman D. J. Lhatoo S. Richerson G. (2016). Sudden unexpected death in epilepsy: epidemiology, mechanisms, and prevention. Lancet Neurol. 15, 1075–1088. 10.1016/S1474-4422(16)30158-2 27571159

[B15] Díaz M. H. Córdova F. M. Cañete L. Palominos F. Cifuentes F. Rivas G. (2015). Inter-channel correlation in the EEG activity during a cognitive problem solving task with an increasing difficulty questions progression. Proc. Comput. Sci. 55, 1420–1425. 10.1016/j.procs.2015.07.136

[B16] Dimitriadis S. I. Salis C. I. Liparas D. (2021). A sleep disorder detection model based on EEG cross-frequency coupling and random forest. medRxiv Preprints. 10.1101/2020.06.10.20126268 33848982

[B17] El-Gindy S. A.-E. Hamad A. El-Shafai W. Khalaf A. A. El-Dolil S. M. Taha T. E.. (2021). Efficient communication and EEG signal classification in wavelet domain for epilepsy patients. J. Ambient. Intell. Human. Comput. 12, 9193–9208. 10.1007/s12652-020-02624-5

[B18] Esmaeili B. Kaffashi F. Theeranaew W. Dabir A. Lhatoo S. D. Loparo K. A. (2018). Post-ictal modulation of baroreflex sensitivity in patients with intractable epilepsy. Front. Neurol. 9:793. 10.3389/fneur.2018.00793 30319527 PMC6168624

[B19] Fisher R. S. Acevedo C. Arzimanoglou A. Bogacz A. Cross J. H. Elger C. E.. (2014). Ilae official report: a practical clinical definition of epilepsy. Epilepsia 55, 475–482. 10.1111/epi.12550 24730690

[B20] Fraiwan L. Lweesy K. Khasawneh N. Wenz H. Dickhaus H. (2012). Automated sleep stage identification system based on time–frequency analysis of a single EEG channel and random forest classifier. Comput. Methods Programs Biomed. 108, 10–19. 10.1016/j.cmpb.2011.11.005 22178068

[B21] Goldenberg M. M. (2010). Overview of drugs used for epilepsy and seizures: etiology, diagnosis, and treatment. Pharma. Therapeut. 35:392. 20689626 PMC2912003

[B22] Gouy-Pailler C. Achard S. Rivet B. Jutten C. Maby E. Souloumiac A.. (2007). Topographical dynamics of brain connections for the design of asynchronous brain-computer interfaces. Annu. Int. Conf. IEEE Eng. Med. Biol. Soc. 2007, 2520–2523. 10.1109/IEMBS.2007.4352841 18002507

[B23] Grigorovsky V. Jacobs D. Breton V. L. Tufa U. Lucasius C. Del Campo J. M.. (2020). Delta-gamma phase-amplitude coupling as a biomarker of postictal generalized EEG suppression. Brain Commun. 2:fcaa182. 10.1093/braincomms/fcaa182 33376988 PMC7750942

[B24] Grosse-Wentrup M. (2008). Understanding brain connectivity patterns during motor imagery for brain-computer interfacing. Adv. Neural Inf. Process. Syst. 21, 561–568. Available online at: https://proceedings.neurips.cc/paper/2008/file/7d04bbbe5494ae9d2f5a76aa1c00fa2f-Paper.pdf

[B25] Gysels E. Celka P. (2004). Phase synchronization for the recognition of mental tasks in a brain-computer interface. IEEE Trans. Neural Syst. Rehabil. Eng. 12, 406–415. 10.1109/TNSRE.2004.838443 15614996

[B26] Hesdorffer D. C. Tomson T. Benn E. Sander J. W. Nilsson L. Langan Y.. (2011). Combined analysis of risk factors for sudep. Epilepsia 52, 1150–1159. 10.1111/j.1528-1167.2010.02952.x 21671925

[B27] Hjorth B. (1970). EEG analysis based on time domain properties. Electroencephalogr. Clin. Neurophysiol. 29, 306–310. 10.1016/0013-4694(70)90143-4 4195653

[B28] Huang M. Wu P. Liu Y. Bi L. Chen H. (2008). “Application and contrast in brain-computer interface between Hilbert-Huang transform and wavelet transform,” in ICYCS '08: Proceedings of the 2008 the 9th International Conference for Young Computer Scientists (Washington, DC: IEEE), 1706–1710. 10.1109/ICYCS.2008.537

[B29] Huang N. E. Shen Z. Long S. R. Wu M. C. Shih H. H. Zheng Q.. (1998). The empirical mode decomposition and the hilbert spectrum for nonlinear and non-stationary time series analysis. Proc. Roy. Soc. Lond. A Math. Phys. Eng. Sci. 454, 903–995. 10.1098/rspa.1998.0193

[B30] Jobson J. (2012). Applied Multivariate Data Analysis, Vol. II, Categorical and Multivariate Methods. New York, NY: Springer Science & Business Media.

[B31] Kaleem M. Guergachi A. Krishnan S. (2021). Comparison of empirical mode decomposition, wavelets, and different machine learning approaches for patient-specific seizure detection using signal-derived empirical dictionary approach. Front. Digit. Health 3:738996. 10.3389/fdgth.2021.738996 34966902 PMC8710482

[B32] Kim Y. Jiang X. Lhatoo S. D. Zhang G.-Q. Tao S. Cui L.. (2020). A community effort for automatic detection of postictal generalized EEG suppression in epilepsy. BMC Med. Inform. Decis. Mak. 20(Suppl. 12):328 10.1186/s12911-020-01306-8 33357232 PMC7758923

[B33] Koley B. Dey D. (2012). An ensemble system for automatic sleep stage classification using single channel EEG signal. Comput. Biol. Med. 42, 1186–1195. 10.1016/j.compbiomed.2012.09.012 23102750

[B34] Kotsiantis S. B. (2007). “Supervised machine learning: a review of classification techniques,” in Proceedings of the 2007 Conference on Emerging Artificial Intelligence Applications in Computer Engineering, Vol. 160, eds I. Maglogiannis, K. Karpouzis, M. Wallace, and J. Soldatos (Amsterdam: IOS Press), 3–24.

[B35] Kuo J. Zhao W. Li C.-S. Kennedy J. D. Seyal M. (2016). Postictal immobility and generalized EEG suppression are associated with the severity of respiratory dysfunction. Epilepsia 57, 412–417. 10.1111/epi.13312 26763069 PMC4783192

[B36] Labate D. La Foresta F. Occhiuto G. Morabito F. C. Lay-Ekuakille A. Vergallo P. (2013). Empirical mode decomposition vs. wavelet decomposition for the extraction of respiratory signal from single-channel ECG: a comparison. IEEE Sensors J. 13, 2666–2674. 10.1109/JSEN.2013.2257742

[B37] Lamichhane B. Kim Y. Segarra S. Zhang G. Lhatoo S. Hampson J.. (2020). Automated detection of activity onset after postictal generalized EEG suppression. BMC Med. Inform. Decis. Mak. 20, 1–10. 10.1186/s12911-020-01307-7 33357222 PMC7758926

[B38] Lhatoo S. Noebels J. Whittemore V. NINDS Center for SUDEP Research. (2015). Sudden unexpected death in epilepsy: identifying risk and preventing mortality. Epilepsia 56, 1700–1706. 10.1111/epi.13134 26494436 PMC4852129

[B39] Lhatoo S. D. Faulkner H. J. Dembny K. Trippick K. Johnson C. Bird J. M. (2010). An electroclinical case-control study of sudden unexpected death in epilepsy. Ann. Neurol. 68, 787–796. 10.1002/ana.22101 20882604

[B40] Lhatoo S. D. Nei M. Raghavan M. Sperling M. Zonjy B. Lacuey N.. (2016). Nonseizure sudep: sudden unexpected death in epilepsy without preceding epileptic seizures. Epilepsia 57, 1161–1168. 10.1111/epi.13419 27221596 PMC5541994

[B41] Li X. Cui L. Tao S. Chen J. Zhang X. Zhang G.-Q. (2017). Hyclasss: a hybrid classifier for automatic sleep stage scoring. IEEE J. Biomed. Health Inform. 22, 375–385. 10.1109/JBHI.2017.2668993 28222004

[B42] Li X. Cui L. Zhang G.-Q. Lhatoo S. D. (2021). Can big data guide prognosis and clinical decisions in epilepsy? Epilepsia 62, S106–S115. 10.1111/epi.16786 33529363 PMC8011949

[B43] Li X. Huang Y. Tao S. Cui L. Lhatoo S. D. Zhang G.-Q. (2019). Seizurebank: a repository of analysis-ready seizure signal data. AMIA Annu. Symp. Proc. 2019, 1111–1120. 32308908 PMC7153150

[B44] Li X. Tao S. Jamal-Omidi S. Huang Y. Lhatoo S. D. Zhang G.-Q.. (2020). Detection of postictal generalized electroencephalogram suppression: random forest approach. JMIR Med. Inform. 8:e17061. 10.2196/17061 32130173 PMC7055778

[B45] Li X. Tao S. Lhatoo S. D. Cui L. Huang Y. Hampson J. P.. (2022). A multimodal clinical data resource for personalized risk assessment of sudden unexpected death in epilepsy. Front. Big Data 5:965715. 10.3389/fdata.2022.965715 36059922 PMC9428292

[B46] Mallat S. (1999). A Wavelet Tour of Signal Processing. Burlington, MA: Elsevier.

[B47] Messaoud R. B. Chavez M. (2021). Random forest classifier for EEG-based seizure prediction. arXiv Preprint. arXiv:2106.04510

[B48] Mier J. C. Kim Y. Jiang X. Zhang G.-Q. Lhatoo S. (2020). Categorisation of EEG suppression using enhanced feature extraction for sudep risk assessment. BMC Med. Inform. Decis. Mak. 20:326. 10.1186/s12911-020-01309-5 33357224 PMC7758934

[B49] Okanari K. Maruyama S. Suzuki H. Shibata T. Pulcine E. Donner E. J.. (2020). Autonomic dysregulation in children with epilepsy with postictal generalized EEG suppression following generalized convulsive seizures. Epilepsy Behav. 102:106688. 10.1016/j.yebeh.2019.106688 31805503

[B50] Omidvar M. Zahedi A. Bakhshi H. (2021). EEG signal processing for epilepsy seizure detection using 5-level Db4 discrete wavelet transform, GA-based feature selection and ANN/SVM classifiers. J. Ambient Intell. Human. Comput. 12, 10395–10403. 10.1007/s12652-020-02837-8

[B51] Orhan U. Hekim M. Ozer M. (2011). EEG signals classification using the K-means clustering and a multilayer perceptron neural network model. Expert Syst. Appl. 38, 13475–13481. 10.1016/j.eswa.2011.04.149

[B52] Oweis R. J. Abdulhay E. W. (2011). Seizure classification in EEG signals utilizing Hilbert-Huang transform. Biomed. Eng. 10, 1–15. 10.1186/1475-925X-10-38 21609459 PMC3116477

[B53] Pachori R. B. (2008). Discrimination between ictal and seizure-free EEG signals using empirical mode decomposition. Res. Lett. Signal Process. 2008:293056. 10.1155/2008/29305621609459

[B54] Petrucci A. N. Joyal K. G. Chou J. W. Li R. Vencer K. M. Buchanan G. F. (2020). Post-ictal generalized EEG suppression and seizure-induced mortality are reduced by enhancing dorsal raphe serotonergic neurotransmission. BioRxiv Preprints. 10.1101/2020.06.28.172460 PMC8713886 33242541

[B55] Redmond S. J. Heneghan C. (2006). Cardiorespiratory-based sleep staging in subjects with obstructive sleep apnea. IEEE Trans. Biomed. Eng. 53, 485–496. 10.1109/TBME.2005.869773 16532775

[B56] Riaz F. Hassan A. Rehman S. Niazi I. K. Dremstrup K. (2015). EMD-based temporal and spectral features for the classification of EEG signals using supervised learning. IEEE Trans. Neural Syst. Rehabil. Eng. 24, 28–35. 10.1109/TNSRE.2015.2441835 26068546

[B57] Rosenow F. Klein K. M. Hamer H. M. (2015). Non-invasive EEG evaluation in epilepsy diagnosis. Expert Rev. Neurother. 15, 425–444. 10.1586/14737175.2015.1025382 25779862

[B58] Seyal M. Hardin K. A. Bateman L. M. (2012). Postictal generalized EEG suppression is linked to seizure-associated respiratory dysfunction but not postictal apnea. Epilepsia 53, 825–831. 10.1111/j.1528-1167.2012.03443.x 22432911

[B59] Smith S. J. (2005). EEG in the diagnosis, classification, and management of patients with epilepsy. J. Neurol. Neurosurg. Psychiatry 76(Suppl 2), ii2–ii7. 10.1136/jnnp.2005.069245 15961864 PMC1765691

[B60] Staba R. J. Stead M. Worrell G. A. (2014). Electrophysiological biomarkers of epilepsy. Neurotherapeutics 11, 334–346. 10.1007/s13311-014-0259-0 24519238 PMC3996122

[B61] Surges R. Strzelczyk A. Scott C. A. Walker M. C. Sander J. W. (2011). Postictal generalized electroencephalographic suppression is associated with generalized seizures. Epilepsy Behav. 21, 271–274. 10.1016/j.yebeh.2011.04.008 21570920

[B62] Theeranaew W. McDonald J. Zonjy B. Kaffashi F. Moseley B. D. Friedman D.. (2017). Automated detection of postictal generalized EEG suppression. IEEE Trans. Biomed. Eng. 65, 371–377. 10.1109/TBME.2017.2771468 29346105 PMC5788043

[B63] Thurman D. J. Hesdorffer D. C. French J. A. (2014). Sudden unexpected death in epilepsy: assessing the public health burden. Epilepsia 55, 1479–1485. 10.1111/epi.12666 24903551

[B64] Vance C. Kim Y. Zhang G. Lhatoo S. Tao S. Cui L.. (2020). Learning to detect the onset of slow activity after a generalized tonic–clonic seizure. BMC Med. Inform. Decis. Mak. 20(Suppl 12):330. 10.1186/s12911-020-01308-6 33357225 PMC7758937

[B65] Vilella L. Lacuey N. Hampson J. P. Rani M. Loparo K. Sainju R. K.. (2019). Incidence, recurrence, and risk factors for peri-ictal central apnea and sudden unexpected death in epilepsy. Front. Neurol. 10:166. 10.3389/fneur.2019.00166 30890997 PMC6413671

[B66] Wei L. Ventura S. Lowery M. Ryan M. A. Mathieson S. Boylan G. B.. (2020). “Random forest-based algorithm for sleep spindle detection in infant EEG,” in 2020 42nd Annual International Conference of the IEEE Engineering in Medicine & Biology Society (EMBC) (IEEE) (Montreal, QC), 58–61. 10.1109/EMBC44109.2020.9176339 33017930

[B67] Wei Q. Wang Y. Gao X. Gao S. (2007). Amplitude and phase coupling measures for feature extraction in an EEG-based brain–computer interface. J. Neural Eng. 4:120–129. 10.1088/1741-2560/4/2/012 17409486

[B68] Worrell G. Gotman J. (2011). High-frequency oscillations and other electrophysiological biomarkers of epilepsy: clinical studies. Biomark. Med. 5, 557–566. 10.2217/bmm.11.74 22003904 PMC3254091

[B69] Wu S. Issa N. P. Rose S. L. Ali A. Tao J. X. (2016). Impact of periictal nurse interventions on postictal generalized EEG suppression in generalized convulsive seizures. Epilepsy Behav. 58, 22–25. 10.1016/j.yebeh.2016.02.025 26994879

[B70] Yang X. Yang X. Liu B. Sun A. Zhao X. (2022). Risk factors for postictal generalized EEG suppression in generalized convulsive seizure: a systematic review and meta-analysis. Seizure 98, 19–26. 10.1016/j.seizure.2022.03.018 35398670

[B71] Zeiler A. Faltermeier R. Keck I. R. Tomé A. M. Puntonet C. G. Lang E. W. (2010). “Empirical mode decomposition-an introduction,” in The 2010 International Joint Conference on Neural Networks (IJCNN), IEEE (Barcelona), 1–8. 10.1109/IJCNN.2010.5596829

[B72] Zhao X. Vilella L. Zhu L. Rani M. Hampson J. P. Hampson J.. (2021). Automated analysis of risk factors for postictal generalized EEG suppression. Front. Neurol. 12:669517. 10.3389/fneur.2021.669517 34046007 PMC8148040

[B73] Zhu C. Kim Y. Jiang X. Lhatoo S. Jaison H. Zhang G.-Q. (2020). A lightweight convolutional neural network for assessing an EEG risk marker for sudden unexpected death in epilepsy. BMC Med. Inform. Decis. Mak. 20(Suppl 12):329. 10.1186/s12911-020-01310-y 33357242 PMC7758925

